# The Role of Video-Assisted Thoracoscopic Surgery in Pediatric Oncology: Single-Center Experience and Review of the Literature

**DOI:** 10.3389/fped.2021.721914

**Published:** 2021-10-12

**Authors:** Giovanna Riccipetitoni, Mirko Bertozzi, Marta Gazzaneo, Alessandro Raffaele, Fabrizio Vatta

**Affiliations:** Department of Pediatric Surgery, Fondazione Istituti di Ricovero e Cura a Carattere Scientifico (IRCCS) Policlinico San Matteo, University of Pavia, Pavia, Italy

**Keywords:** thoracoscopy, pediatrics, oncology, mini-invasive surgery, robotic-assisted thoracoscopic surgery

## Abstract

**Aim:** Video-assisted thoracoscopic surgery (VATS) has been widely used in the last decades. Nevertheless, the pros and cons of thoracoscopy vs. open surgery in pediatric oncology are still under debate. In literature, VATS has been applied for both diagnostic and ablative surgery to treat neurogenic tumors, thymic neoplasms, lung tumors and metastases, germ cell tumors, lymphoproliferative diseases, and other rare tumors. Recent reviews described excellent outcomes in pediatric oncology as well as in the treatment of adult lung cancer, with a significantly higher rate of mortality and complication in thoracotomy compared to VATS. We reviewed our experience on thoracoscopy in pediatric malignancy and compared it to the literature.

**Materials and Methods:** This was a retrospective cohort-study of pediatric oncological patients who underwent VATS at our institution from 2007 to 2020, and a review of the recent literature on the topic.

**Results:** A total of 43 procedures were performed on 38 oncological patients (18 males, 20 females). Median age was years 7.72 (0.35–18.6). Diagnosis: 10 neurogenic tumors, nine hematological diseases, five metastases, four lypoblastomas, three thymic pathologies, three germ cell tumors, two pleuropneumoblastomas, two myofibroblastic tumors, one myoepithelial carcinoma, one liposarcoma, and three suspected oncological mass. In three cases, a 3D model was elaborated to better plan the surgical approach. Diagnostic biopsies were 22 (51.1%), and ablative surgeries, 21 (48.9%). One neurogenic tumor was resected with the Da Vinci Robot. Median operative time was 120 min (30–420). A drain was left in place in 20 (46.5%) for a median of 4 days. Median length of hospitalization was 5 days (1–18). One case (2.3%) was converted (intraoperative bleeding). There were three post-operative complications (7.0%): one pneumonia, one pleural effusion, and one diaphragmatic paralysis (need for plication). Results were compared to recent literature, and morbidity and conversion rate were comparable to reviewed publications.

**Conclusion:** VATS represents a valuable tool for diagnostic and therapeutic procedures in pediatric oncology. Nonetheless, it is a challenging technique that should be performed by expert surgeons on oncological and mini-invasive surgery. Three-dimensional reconstruction can optimize the pre-operative planning and guarantee a safer and more targeted treatment. Finally, the advent of robotics-assisted surgery represents a new challenge that may further implement the advantages of VATS.

## Introduction

Minimally invasive surgery (MIS), including thoracoscopy, has been widely applied in the last few decades and has now become the gold standard approach for a variety of procedures for both adults and children (1–3).

The advantages of MIS compared to the open approach are well-known, as it reduces tissue trauma, decreases post-operative pain, shortens hospital stay, and guarantees better cosmetic and long-term functional results (4, 5). Moreover, among of the most important benefits of thoracoscopy are the virtually non-existing musculoskeletal complications such as chest wall deformities, rib fusion, shoulder girdle weakness, and scoliosis, which can occur in up of 30% of pediatric patients undergoing thoracotomy (6, 7).

Nevertheless, video-assisted thoracoscopic surgery (VATS) still represents a challenge due to the limitation of working space, the smaller body size of children, reduced tactile feedback, and the lack of three-dimensional vision.

As far as it concerns pediatric oncology, in the last decades, many reports and experiences have been published on the use of thoracoscopy to diagnose and resect intrathoracic neoplasms. Nonetheless, the role of VATS is still considered a developing field and no consensus exists regarding the details of its application. Specific limitations are an even lower exposure in pediatric oncological cases compared to general thoracoscopic pathologies and the inability to perform digital palpation.

In the last years, robotics has been applied to perform a wide variety of procedures, including thoracic surgery (8). Nonetheless, very few reports describe the use of robotics in pediatric oncological thoracic surgery and its use is not yet validated.

The aim of this study is to review our experience in VATS and robotics-assisted thoracoscopy in pediatric malignancy and to compare it to the most recent literature.

## Materials and Methods

We performed a retrospective review of all pediatric oncological patients who underwent video-assisted thoracoscopic surgery (VATS) and robotics-assisted thoracoscopic surgery (RATS) at our Institution from 2007 to 2020.

Patients over 20 years old and/or with thoracic pathologies treated with thoracotomy or endoscopic procedures were excluded.

We analyzed demographic data, including age at surgery, sex, pathology, and possible comorbidities; operation time (OT); length of hospital stays (LHS); perioperative complications; and post-operative outcomes. All complications were classified according to the Clavien–Dindo classification and graded from I to V. If present, length of chest drain aspiration was evaluated.

A specific search was performed in scientific database (PUBMED, MEDLINE, and EMBASE) to compare our experience with the most recent literature on the field. We selected articles reporting thoracoscopy in oncological pediatric patients between 2014 and March 2021 using the following key-words: (pediatric) or (children) and (thoracoscopy) or (VATS) and (oncology) or (tumor).

Inclusion criteria were:

- Articles published between January 2014 and March 2021
- Articles written in English
- Articles focusing on VATS in pediatric oncology
- Median/mean age <18 years old
- Case series with more than 10 patients
- Articles where data concerning demographics, surgical indications, complication rate, and conversion rate were clearly deductible

All data were elaborated using the statistical software “R”, version 3.4.1.

Descriptive statistics was used to present findings, and quantitative variables were expressed as median (range) to express our data. Data elaborated from the literature review were expressed as median (range) or mean ± standard deviations depending on the reference found in the original articles.

### Surgical Methods

Under general anesthesia, lung collapse has been achieved by single-lung ventilation. We routinely used a double lumen endotracheal tube in adolescent patients, mainly for ablative procedures. In small children (more than 6 months of age), we adopted a standard endotracheal intubation associated to the use of an endobronchial blocker. Low-pressure CO_2_ insufflation (4–5 mmHg; flow 0.5 L/min) was employed in infants <6 months or when a quick biopsy of anterior mediastinal tumors was required. NIRS and BIS brain-monitoring were applied according to the age of the patients.

Patients were placed in lateral decubitus, with an angle ranging from 60 and 90° to 120°, depending on the mass localization.

A 10-mm one-trocar operative optic was employed for biopsy in three cases (one germ cell tumor, one pleuropneumoblastoma, and one myofibroblastic inflammatory tumor).

A three-trocar technique was adopted in most of the remaining cases, positioned according to the characteristics of the lesion. The optic (5 or 10 mm) was placed in the typical mid-axillary position under the shoulder angle, whereas the operative trocars were placed in a triangulation fashion anteriorly or posteriorly depending on the site of the mass.

In small children, a 3-mm operative trocar technique with 5-mm optic was carried out to perform biopsies, whereas in ablative surgery, one 5-mm operative trocar was reserved for Ligasure® or other vessel sealers. In recent cases, a 3-mm vessel sealer has been used, allowing a 3-mm operative VATS also for ablative procedures.

Pulmonary resection was safely performed with staplers (5 or 12 mm), which required an additional trocar. Specimens were retrieved using a 5 mm Endobag.

Regarding the use of chest drain, we tried to avoid drainage placement in diagnostic biopsies, except in case of complications such as intraoperative bleeding. A drain was normally applied at the end of any ablative surgery.

When possible, an early extubation of the patients was made a few hours after the VATS procedure.

About RATS, we performed one procedure using the Da-Vinci SI® Robot for a mass located on the supero-posterior mediastinum. Optic trocar was positioned in the sixth intercostal space on the midaxillary line. Operative trocars were positioned 8 cm away from the optic, in the fifth intercostal space on the anterior axillary line, and in the seventh intercostal space on the paravertebral line, respectively. Finally, a 5-mm auxiliary trocar was placed in the fourth intercostal space on the anterior axillary line.

## Results

A total of 43 procedures were performed on 38 oncological patients (18 males, 20 females). Median age was 7.72 (0.35–18.6). Patients were grouped according to diagnosis, and we observed high prevalence of neurogenic tumors and hematologic diseases, as shown in Table 1.

**Table 1:** Summarized data of all oncological patients undergoing VATS (in chronological order).

ID^*^	**Sex**	**Year**	**Age (y)**	**Pre-op Imaging**	**Diagnosis**	**Localization**	**Side**	**Intervention**	**Operative Time (min)**	**Peri-op compli-cations**	**Conversion**	**Drain (days)**	**LHS (days)**	**Post-op complications**	**Type of complication (Clavien Dindo Classification)**
JM	M	2007	14.00	TC	Germ cell tumor	Complete invasion of all hemithorax	R	Biopsy (1 trocar)	30	No	No	No	2	No	
VG	M	2007	14.00	TC	T-cell lymphoma	Anterior mediastinum	L	Biopsy	60	No	No	No	3	No	
BL	F	2008	3.00	TC	Germ cell tumor	Complete invasion of all hemithorax	L	Biopsy	90	Yes (bleeding)	No	Yes (4)	7	No	
GD	M	2009	1.00	TC	Pleuropulmonary blastoma	Superior and medium lobe	R	Biopsy (1 trocar)	60	No	No	Yes (3)	4	No	
AL	F	2010	8.07	TC	Suspicion of renal carcinoma metastasis (not confirmed at histology)	Inferior lobe	L	Biopsy	105	No	No	No	14	No	
BA	M	2010	3.94	TC	Nephroblastoma metastasis	Inferior lobe	L	Mass excision (atypical pulmonary excision)	155	No	No	No	2	No	
ML (1)	M	2010	18.16	TC	Casteman's disease	Anterior mediastinum	R	Biopsy	75	No	No	No	1	No	
PC	F	2010	4.79	TC	Ganglioneuroma metastasis	Costophrenic recess	L	Mass excision	180	No	No	No	3	No	
ML (2)	M	2011	18,65	TC	Casteman's disease	Anterior mediastinum	R	Mass excision	330	No	No	No	2	No	
CA	F	2012	0.50	TC MRI	Neuroblastoma	Superior mediastinum	L	Biopsy	90	No	No	No	3	No	
CS	F	2010	14.11	TC	Diffuse large B-cell lymphoma	Antero-Superior mediastinum	R	Biopsy	80	No	No	No	–^**^	No	
HL	M	2012	16.53	TC	Neuroblastoma	Posterior mediastinum	R	Biopsy	60	No	No	No	3	No	
DM	M	2012	11.04	TC	Precursor T-cell lymphoblastic lymphoma	Retrosternal region	R	Biopsy	150	No	No	No	–^**^	No	
PG	M	2012	0.35	TC	Cystic lymphangioma	Superior mediastinum	R	Mass excision	165	No	No	Yes (4)	2	Yes	Diaphragmatic paralysis, need for laparoscopic diaphragmatic plication (IVa) 1 year later
BL	M	2014	6.00	TC	Classical Hodgkin's lymphoma	Anterior mediastinum	R	Biopsy	70	No	No	No	3	No	
BR	F	2014	10.00	MRI	Ganglioneuroblastoma	Posterior mediastinum	L	Mass excision	120	No	No	Yes (3)	5	No	
SR	F	2019	1.25	MRI	Lipoblastoma (recidive)	Lateral chest wall, medullar invasion	R	Mass excision	240	No	No	Yes (7)	–^**^	No	
FE (1)	F	2014	1.19	TC	Neuroblastoma	Antero-Superior mediastinum	R	Biopsy	180	No	No	No	5	No	
FE (2)	F	2014	1.84	TC MRI	Neuroblastoma	Antero-superior mediastinum	R	Biopsy	90	No	No	No	2	No	
GD	M	2014	13.61	TC	Osteosarcoma metastasis	Inferior lobe	L	Mass excision	90	No	No	No	2	No	
PI	F	2014	10.00	TC MRI	Ganglioneuroblastoma	Posterior mediastinum	L	Mass excision	120	No	No	Yes (3)	18	Yes	Pleural effusion after drain removal, need for thoracic drain re-insertion for 7 days (IIIb)
RG	F	2014	11.96	TC MRI	Myofibroblastic inflammatory tumor	Pulmonary hilum	R	Biopsy	250	No	No	Yes (4)	7	No	
VA	M	2014	16.13	TC	Germ cell tumor	Costophrenic recess	R	Biopsy	115	No	No	No	11	Yes	Post-op pneumonia (II)
AA (1)	F	2015	7.67	TC	Suspicion of neoplastic mass (not confirmed at histology)	Middle mediastinum	R	Biopsy	95	No	No	No	5	No	
AA (2)	F	2015	7.72	TC	Suspicion of neoplastic mass (not confirmed at histology)	Middle mediastinum	R	Biopsy	105	No	No	No	2	No	
AA (3)	F	2015	8.18	TC	Suspicion of neoplastic mass (not confirmed at histology)	Middle mediastinum	R	Biopsy + Culture test	110	No	No	No	3	No	
LAn	M	2015	15.77	TC	Classical Hodgkin's lymphoma	Costophrenic recess	L	Biopsy	160	No	No	No	2	No	
PB	M	2015	5.44	TC	Nephroblastoma metastasis	Superior lobe	R	Mass excision (atypical pulmonary excision)	350	Yes (bleeding)	Yes	Yes (4)	7	No	
RA	F	2015	10.00	MRI	Thymic teratoma	Anterior mediastinum	L	Mass excision (thymectomy)	200	No	No	Yes (5)	7	No	
ZS	M	2015	7.00	MRI	Thymic teratoma	Anterior mediastinum	R	Mass excision (thymectomy)	195	No	No	Yes (4)	6	No	
TM	F	2016	11.00	MRI	Thymoma	Anterior mediastinum	R	Mass excision (thymectomy)	220	No	No	Yes (5)	7	No	
TL	F	2017	16.47	TC MRI	Pleomorphic liposarcoma	Costophrenic recess	R	Biopsy	110	No	No	No	3	No	
BB	F	2018	7.00	TC MRI	Neuroblastoma	Posterior mediastinum	L	Mass excision	190	No	No	Yes (3)	5	No	
BF	M	2018	12.00	MRI	Lipoblastoma	Lateral chest wall	R	Mass excision	180	No	No	Yes (5)	7	No	
EZ	F	2018	6.00	TC MRI	Myofibroblastic inflammatory tumor	Superior and medium lobe, trachea and heart invasion	R	Biopsy (1 trocar)	60	No	No	No	3	No	
LAr	M	2018	2.00	TC MRI	Neuroblastoma	Posterior mediastinum	L	Mass excision	119	No	No	Yes (4)	6	No	
LM	M	2018	7.00	TC MRI	Cystic lesions after pleuropulmonary blastoma excision	Superior, medium, and inferior lobe (3 different lesions)	R	Masses excision (atypical pulmonary excision)	190	No	No	Yes (2)	7	No	
LN	F	2018	7.00	TC MRI	Myoepithelial carcinoma	Superior and inferior lobe, heart invasion	L	Biopsy (with associated bronchoscopic biopsy)	80	No	No	No	7	No	
BG	F	2019	5.00	MRI	Lipoblastoma	Posterior mediastinum	L	Mass excision	150	No	No	Yes (4)	6	No	
BM	F	2020	13.28	TC MRI	Lipoblastoma	Superior mediastinum	R	Mass excision	305	No	No	Yes (7)	6	No	
FE (3)	F	2020	7.59	MRI	Ganglioneuroblastoma intermixed	Anterior mediastinum	R	Robot-Assisted mass excision	290	No	No	Yes (6)	7	No	
PD	M	2020	14.80	TC	Classical Hodgkin's lymphoma	Anterior mediastinum	R	Mass excision (thymectomy)	285	No	No	Yes (3)	5	No	
SL	F	2020	7.00	TC MRI	Immature teratoma metastases	Superior, medium and inferior lobe (6 different lesions)	R	Mass excision (multiple lesions)	420	No	No	Yes (7)	8	No	

* *If in the same patient, a different number of procedures is specified within brackets*.

** *LHS was not considered as they started further therapy during the same hospitalization*.

We performed diagnostic biopsies in 22 cases (51.1%), compared to 21 cases of ablative surgeries (48.9%); 42 out of 43 procedures were completed by VATS technique (97.7%); only one patient required an open conversion (2.3%).

The procedures were carried out by single-lung ventilation using a double-lumen endotracheal tube in 14 adolescent patients, and standard endotracheal intubation associated to the use of an endobronchial blocker was adopted in 16 cases. In the remaining 13 cases, lung collapse was obtained with low-pressure CO_2_ insufflation.

One patient with growing ganglioneuroblastoma intermixed of the supero-posterior mediastinum in a 7-years-old girl with Horner's syndrome and positive image-defined risk factor (IDRF) (symptomatic encased subclavian vessels) was operated with the Da-Vinci SI® Robot-assisted thoracoscopic surgery. The robotic approach was chosen to achieve a safer and more precise dissection.

In the whole series, median OT was 120 min (30–420): 90 for diagnostic biopsies, 190 for ablative surgery.

Overall, a thoracic drain was left in place in 20 cases, for a median length of 4 days.

Only three diagnostic procedures required chest drainage due to intraoperative bleeding (one germ cell tumor), pneumothorax (one pleuropneumoblastoma), and pleural effusion (one myofibroblastic inflammatory tumor). As for ablative surgeries, a chest tube was positioned in 17 out of 21 procedures.

Median LHS was 5 days (1–18).

In two cases, we observed intraoperative bleeding (4.7%): one biopsy of a germ cell tumor managed thoracoscopically and one lung resection for nephroblastoma metastasis which required conversion to open surgery. The conversion rate to open surgery was 2.3% in the whole series.

In our series, we observed three post-operative complications (7.0%): one pneumonia (Clavien Dindo: grade II) 2 weeks after biopsy for a germ cell tumor; one pleural effusion a week after resection of a posterior mediastinal ganglioneuroma, which required a re-insertion of a thoracic drain for a further 7 days (IIIb); and one persistent right diaphragmatic paralysis after the excision of a giant cystic lymphangioma, treated with a laparoscopic diaphragmatic plication (IVa) 1 year later, which required intensive care assistance.

No tumor upstaging or trocar site recurrences occurred.

An advanced 3D virtual reconstruction and printing technology was recently applied in three complex cases (multiple immature teratoma metastases, multiple cystic lesions after pleuro-pneumoblastoma excision, vessel encasement in IDRF-positive ganglioneuroblastoma intermixed).

A review of the most recent literature on the topic was performed. From a total of 229 articles, seven were included in the review according to the inclusion criteria (Table 2) (9–15).

**Table 2:** Review of the most recent literature.

**References**	**Year**	**Study**	**Number**	Indication^*^	**Median age (y)**	Types tumor^*^	**LHS (days)**	**Conversion**	Complications^**^	**Port-Site metastases**	**Intraoperative rupture**
Lautz et al. (9)	2021	Retrospective	48	AS	13.9 ± 4.5	Metastatic osteosarcoma	2	0%	2.1% SSI; 2.1%; DVT or PE 6.3%	0	0
Abdelhafeez et al. (10)	2019	Retrospective	179	B (81.6%) AS (18.5%)	NA	B: 113 Pulmonary lesions, 30 Mediastinal tumors, 3 Pleural lesions; AS: 21 Pulmonary metastases, 7 Neurogenic tumors, Other: 5	NA	15.1%	7.8% (B: 10 PNX, AS: 3 PNX, 2 bleeding, 1 SSI, 1 other)	0	0
Da et al. (12)	2019	Retrospective	43	AS	3.7 ± 2.9	13 Neurogenic tumors, 5 Cystic Teratoma, 4 Lymphangioma, Other: 21	10.0 ± 5.5	0%	4.7% (not precisely specified)	0	0
McDaniel et al. (15)	2018	Retrospective	35	AS	11.25 (0.67–23.50)	6 Osteosarcoma, 5 Ewing sarcoma, 5 Hepatoblastoma, 3 Rhabdomyosarcoma, 3 Synovial cell Sarcoma, Other: 13	NA	0%	17.0% (6 PNX)	0	0
Acker et al. (13)	2015	Retrospective	77	B (72.3%) AS (27.7%)	10.7 ± 6.3	B: Lymphoma 21, Metastases 29, Other: 6 AS: Neuroblastoma 11, Metastatic disease 9, schwannoma 1	5.9 (±8.1)	5.2%	11.0% (not precisely specified)	0	0
Sato et al. (11)	2016	Retrospective	21	AS	6.9 ± 4.6	11 Neurogenic tumors, 6 Germ Cell Tumors, Other: 4	8.0 ± 3.7	0%	20% (1 brachial plexus palsy, 1 Horner's syndrome, 1 atelectasis, 1 palsy of upper limb)	0	0
Irtan et al. (14)	2015	Retrospective	20	AS	3.3 (0.3–11.6)	Neuroblastic tumors	5.2 (2–10)	15%	20.0% (3 Chlyothorax, 1 Horner's syndrome)	0	0
Riccipetitoni et al. (2021)	2021	R	43	B (51.1%) AS (48.9%)	7.72 (0.35–18.6).	10 Neurogenic tumors, 9 Hematological diseases, 5 Metastases, 4 Lypoblastomas, 3 thymic pathologies, 3 germ-cell tumors, Other: 9 (see Table 1)	5 (1–18)	2.3%	7.0% (see Table 1)	0	0

* *B, biopsy; AS, ablative surgery*.

** *SSI, surgical site infection; DVT, deep venous thrombosis; PE, pulmonary embolism; PNX, pneumothorax*.

All papers were retrospective reviews and analyzed VATS to perform either tumor biopsy, ablative surgery, or both. A total of 423 children were included in the review, with a median age of 8.8 years. Among pulmonary lesions, metastases resulted as the most common indication for surgery. On the other hand, in case of a mediastinal lesion, neurogenic tumor was the most represented group. Conversion rate ranged from 0 to 15.1%, most commonly due to intraoperative bleeding or difficult dissection of the mass due to strong adhesions. Complications occurred in 2.1–20% of cases, pneumothorax being the most common post-operative one. No articles reported port-site recurrences, intraoperative tumor rupture, or tumor upstaging. Average LHS ranged from 2 to 10 days.

## Discussion

VATS in pediatric oncological surgery represents a great surgical achievement but still faces many challenges. Alongside the limitations of the technique, there are no guidelines on the details of the use of VATS or its contraindications. However, its application is expanding, and the experience reported in literature is growing.

Our study presents a large cohort of oncological patients undergoing VATS and proves the feasibility and effectiveness of the use of MIS in pediatric oncology; 97.7% of cases in our series were successfully treated by thoracoscopy.

As far as it concerns the use of VATS for diagnostic procedures, MIS has been effectively used to perform mediastinal mass biopsies as well as pulmonary masses (10, 13, 16–18). Reported advantages of thoracoscopy are the possibility of exploring the entire surface of the lung and pleura, and performing multiple biopsies if required (e.g., germinal tumors). Morbidity, mortality, and length of hospitalization are lower when compared to open surgery (19). Success rate of thoracoscopic-assisted biopsies have been reported in literature ranging from 96.7 to 100% for both histological and/or bacteriological diagnosis (12, 19–28). Nonetheless, different techniques have been proposed to further enhance the accuracy of MIS, such as intraoperative ultrasound localization (both trans-pleural and endoscopic) (29), CT-guided needle localization with methylene blue staining, or micro-coil application (15, 30, 31).

Our experience confirms the feasibility of VATS for diagnostic procedures. Among our cases, we performed three one-trocar site procedures, without complications. Nevertheless, indication for this technique in pediatrics is limited to procedures with low risk of bleeding.

**Figure 1:**
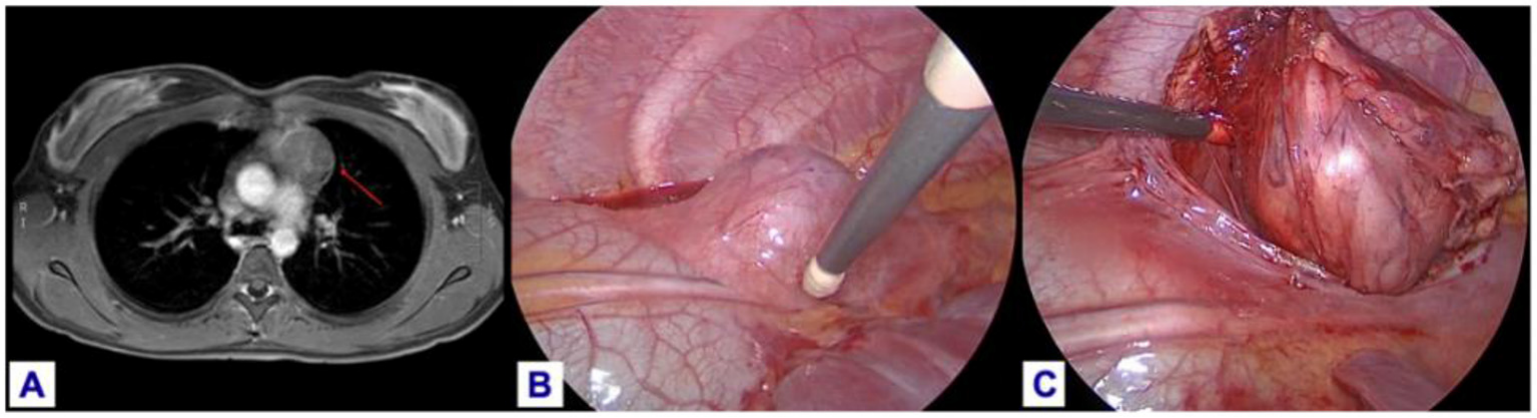
Thymoma excision in an 11-year-old girl: **(A)** MRI pre-operative imaging. **(B)** Identification and preservation of the phrenic nerve. **(C)** Dissection of the thymus from the pericardium.

Thoracoscopic surgery has been largely applied to excise mediastinal malignancies, and its use has been extended in pediatrics as well. Although in our cohort we did not record conversion to open surgery, in the most recent literature, conversion rate has been reported in up to 15% of cases (10–14) (Figure 1).

When performing mediastinal mass excisions (*n* = 14), we positioned a chest drain in 85.7% of patients. Nonetheless, its use is not mandatory, and in literature, chest drainage is not performed in up to 35% of procedures (29–31). A recent review of 2021 by Yu-Wei Liu reported how eliminating chest drain placement after mediastinal tumor resection can decrease post-operative pain and hospital stay without increasing complications or compromising patient safety (32).

In pediatrics, the most common mediastinal neoplasms are neurogenic tumors (neuroblastoma, ganglioneuroblastoma, and ganglioneuroma). Surgical approach has been evolving, and their resection is now often performed thoracoscopically, guaranteeing the well-known advantages of MIS (30). Different studies have found similar oncological outcomes and comparable rates of complication between the MIS and open approaches (30, 31, 33). Nonetheless, at least one port-site dissemination has been described (34). To date, VATS for neurogenic tumors does not yet have specific guidelines, although there are no contraindications. Nonetheless, literature still reports the frequent use of open approach in case of a large mediastinal tumor (35). In our series, seven patients with neurogenic tumor underwent a total of 10 procedures (four biopsies and six mass excisions). Among the excision group, five patients were submitted to a thoracoscopic surgery and one patient to a robotics-assisted procedure. All children presented IDRF-negative tumors except the case treated by RATS.

Pre-operative diagnostic workup and identification of IDRFs obtained by computed tomography (CT) and/or magnetic resonance are essential to precisely localize the lesion and identify the extent of the disease. A recent systematic review conducted by the APSA Cancer Committee confirmed how a pre-operative objective assessment by IDRFs and size criteria are recommended to guide the approach, in order to follow oncologic principles of surgical resection of neuroblastic tumors with the least possible morbidity (36).

**Figure 2:**
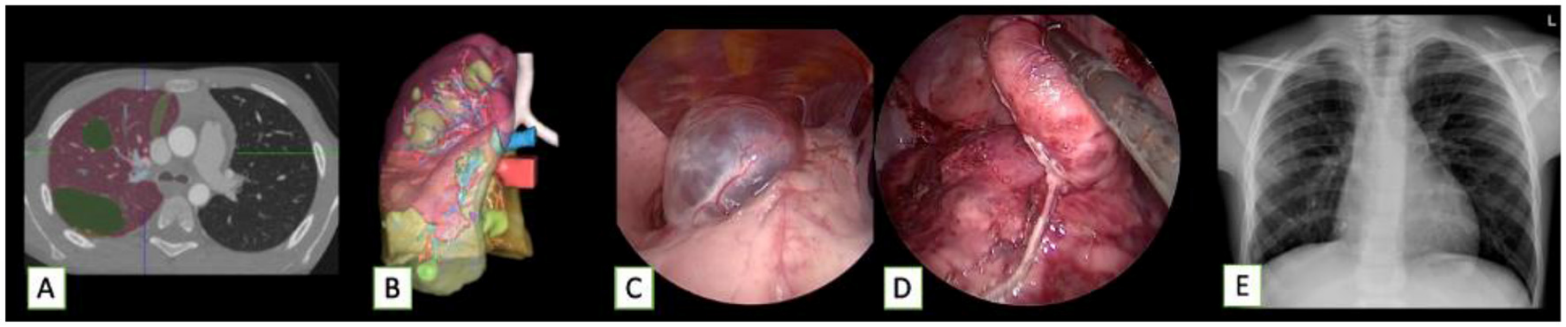
Cystic lesions after surgery and chemotherapy for a pleuropulmonary blastoma in a 7-year-old boy, who then underwent MIS excision of multiple masses. **(A,B)** Reconstruction and 3D model of the lesions. **(C,D)** Intraoperative thoracoscopic view. **(E)** Post-operative X-ray.

**Figure 3:**
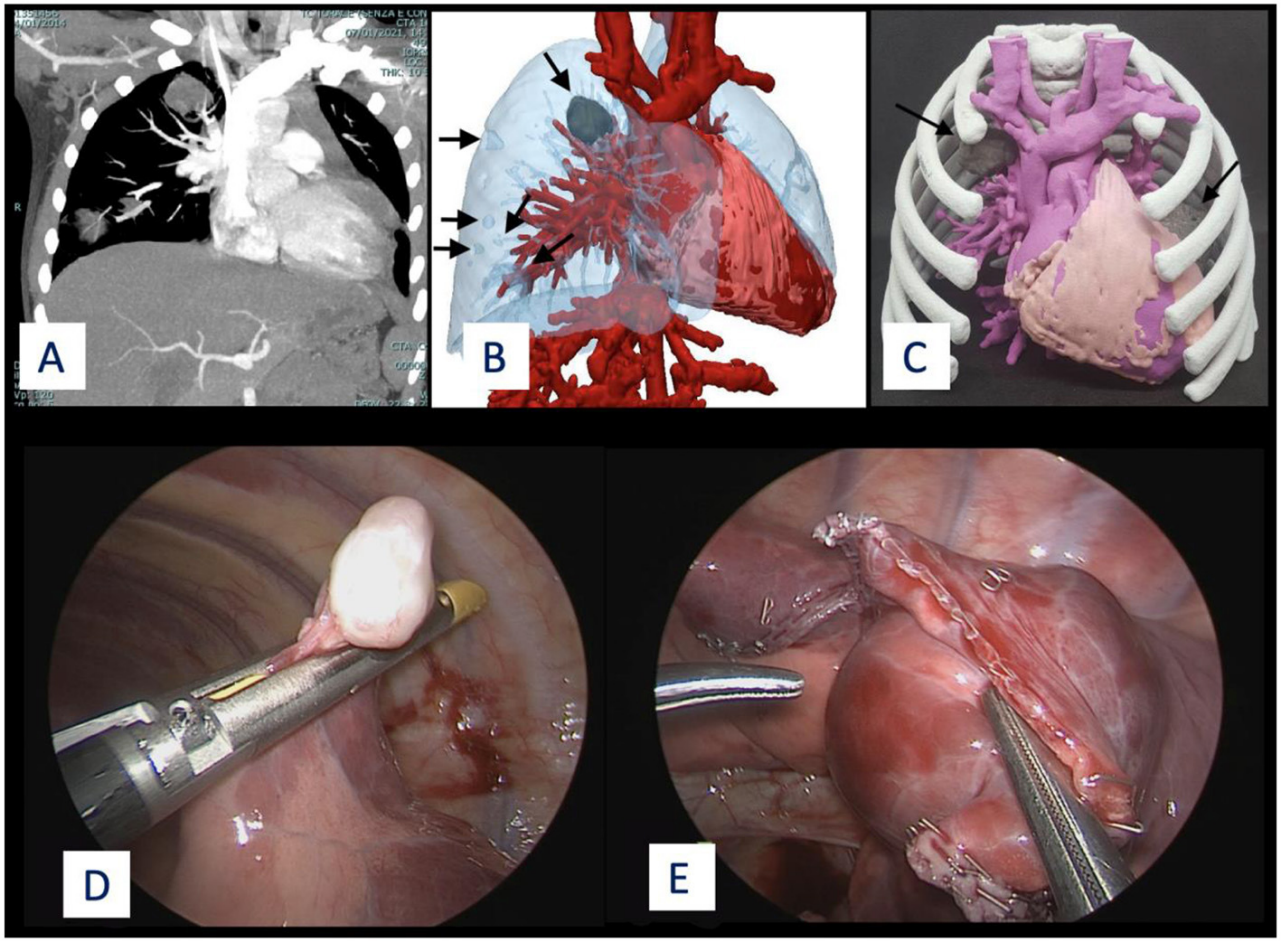
Multiple metastases of immature teratoma in a 7-year-old girl. Left single metastasis was excised through an open procedure, due to dimension and site of the mass. The remaining lesions (*n* = 6, one solid, five cystic ones) on the right lobe were excised through VATS. **(A)** CT image. **(B)** 3D virtual reconstruction. **(C)** 3D printed model showing the two solid lesions (colored in gray). **(D,E)** Intraoperative view.

In recent years, 3D virtual reconstruction and printing technologies have been increasingly applied to further implement the possibility of a more precise surgical planning (37, 38). These technologies enable surgeons to simulate beforehand the surgical procedure, potentially reducing the risk of intraoperative complications and allowing a conservative surgery when indicated (39–41) (Figures 2 and 3). In our experience, these techniques were chosen in complex cases with multiple lesions and vessel encasement and allowed the formulation of a personalized surgical strategy.

VATS is largely used to perform segmentectomies and lobectomies for pulmonary lesions in oncological adult patients. A recent meta-analysis reviewed 34 studies (183,426 patients), comparing open-approach, VATS, and robotics-assisted surgery. Their results showed how MIS seemed safer compared to open surgery, with reduced 30-day mortality, pulmonary complications, and overall complications with equivalent oncologic outcomes and 5-year overall survival (42). Nevertheless, use of VATS in pediatric lung tumors is not yet validated, and only small case-series can be found.

As far as it concerns metastatic disease, osteosarcoma represents one of the most relevant causes of metastases in children. Surgical management of osteosarcoma is still strongly debated, knowing that the most important prognostic indicator for this disease is the complete resection of all lesions (43). One of the main disadvantages of VATS is the impossibility to perform digital palpation to detect possible lesions not identified at imaging. Compared to other neoplasia, the higher calcification of osteosarcoma metastases allows for manual palpation at sizes below the resolution of CT. Nonetheless, many centers have started using thoracoscopy for osteosarcoma metastases due to the acknowledged advantages of MIS compared to open thoracotomy. A recent collaborative study on 202 patients showed that, in patients with limited metastases, both mortality and risk of pulmonary recurrence were similar when comparing VATS and thoracotomy (44). The risk of possible port-site metastases should not be neglected, as it has already been reported in the case of an 18-year-old female who presented with port site metastases within 4 months of pulmonary metastasectomy for osteosarcoma (45).

In the last decades, robotics-assisted surgery (RAS) has been increasingly employed in several complex procedures, and its use is now standardized for many interventions. The known advantages are the 3D vision, seven degrees of freedom, tremor filtration, and precise camera control (46). In pediatrics, its usage has more limitations due to the mismatch between the robotic arms and trocar dimensions compared to small children, the absence of haptic feedback, and the potential risk of tumor rupture and spillage. No guidelines or recommendations have yet been proposed from the main association of pediatric surgery in this field (47). Despite this, RAS is starting to be implemented in this field as well. In a recent review of 2020 (48), a total of 13 thoracic oncological neoplasias were treated (thymoma, neuroblastoma, inflammatory myofibroblastic tumor, bronchial carcinoid tumor). No tumor recurrence or port-site metastases have been registered; two post-operative pneumothoraxes occurred, and one conversion due to difficult dissection of a neuroblastoma was recorded (48). In our single case, we did not observe any robot-related complications such as adjacent organ injury, positioning-related injury, or injury due to robotic arms. Despite some promising results of RAS reported in literature, its use should be reserved to highly selected cases and, due to lack of recommendations, surgical indication should be discussed by an expert multidisciplinary team (48–50).

## Limitations of the Study

We should recognize some limitations of the study, related to the predictable low case-volume and heterogeneity of rare diseases that we treated. The analysis of the recent literature faces the same challenge. In addition, the comparison of MIS to open surgery may be problematic in ascertainment with observational studies due to potential unmeasured selection bias, difficulty of retrospective patient-level comparison, and lack of randomized trials (10, 51).

## Conclusion

VATS has now become a standardized tool in pediatric surgery. From our experience, it may be applied effectively in thoracic pediatric oncology for both diagnostic and therapeutic purposes with low complication and conversion rate.

It is a challenging procedure that requires a dedicated multidisciplinary team with competencies in pediatric oncology, radiology, anesthesiology, and mini-invasive thoracic surgery.

Pre-operative patient selection requires a strict adherence to tumor protocols; IDRF evaluation; and assessment of biology, stage, and dimension of the tumor. Recent technologies such as 3D virtual reconstruction and 3D printing may optimize this selection and guarantee a safer and more targeted treatment. The role of robotics-assisted surgery needs to be defined.

## Data Availability Statement

The original contributions presented in the study are included in the article/supplementary materials, further inquiries can be directed to the corresponding author/s.

## Ethics Statement

Ethical review and approval was not required for the study on human participants in accordance with the local legislation and institutional requirements. Written informed consent to participate in this study was provided by the participants' legal guardian/next of kin. Written informed consent was obtained from the minor(s)' legal guardian/next of kin for the publication of any potentially identifiable images or data included in this article.

## Author Contributions

GR contributed to conception and design of the study. FV organized the database and performed the statistical analysis. FV, GR, and MB wrote the first draft of the manuscript. FV, MB, and GR wrote sections of the manuscript. MG and AR help the revision process and implemented sections of the manuscript. All authors contributed to manuscript revision, read, and approved the submitted version.

## Conflict of Interest

The authors declare that the research was conducted in the absence of any commercial or financial relationships that could be construed as a potential conflict of interest.

## Publisher's Note

All claims expressed in this article are solely those of the authors and do not necessarily represent those of their affiliated organizations, or those of the publisher, the editors and the reviewers. Any product that may be evaluated in this article, or claim that may be made by its manufacturer, is not guaranteed or endorsed by the publisher.

## References

[1] Lacher M Kuebler JF Dingemann J Ure BM. Minimal invasive surgery in the newborn: current status and evidence. Semin Pediatr Surg. (2014) 23:249–56. 10.1053/j.sempedsurg.2014.09.004 25459008

[2] Ponsky TA Rothenberg SS. Minimally invasive surgery in infants less than 5 kg: experience of 649 cases. Surg Endosc. (2008) 22:2214–9. 10.1007/s00464-008-0025-7 18649102

[3] Ure BM Schmidt AI Jesch NK. Thoracoscopic surgery in infants and children. Eur J Pediatr Surg. (2005) 15:314–8. 10.1055/s-2005-865793 16254842

[4] Lawal TA Gosemann JH Kuebler JF Glüer S Ure BM. Thoracoscopy versus thoracotomy improves midterm musculoskeletal status and cosmesis in infants and children. Ann Thorac Surg. (2009) 87:224–8. 10.1016/j.athoracsur.2008.08.069 19101302

[5] Yang YF Dong R Zheng C Jin Z Chen G Huang YL. Outcomes of thoracoscopy versus thoracotomy for esophageal atresia with tracheoesophageal fistula repair. Medicine. (2016) 95:e4428. 10.1097/MD.0000000000004428 27472740 PMC5265877

[6] Jaureguizar E Vazquez J Murcia J Diez Pardo JA. Morbid musculoskeletal sequelae of thoracotomy for tracheoesophageal fistula. J Pediatr Surg. (1985) 20:511–4. 10.1016/S0022-3468(85)80477-2 4057018

[7] Korovessis P Papanastasiou D Dimas A Karayannis A. Scoliosis by acquired rib fusion after thoracotomy in infancy. Eur Spine J. (1993) 2:53–5. 10.1007/BF00301057 20058450

[8] Durand M Musleh L Vatta F Orofino G Querciagrossa S Jugie M. Robotic lobectomy in children with severe bronchiectasis: a worthwhile new technology. J Pediatr Surg. (2021) 56:1606–10. 10.1016/j.jpedsurg.2020.11.009 33250217

[9] Lautz TB Farooqui Z Jenkins T Heaton TE Doski JJ Cooke-Barber J. Thoracoscopy vs thoracotomy for the management of metastatic osteosarcoma: a pediatric surgical oncology research collaborative study. Int J Cancer. (2021) 148:1164–71. 10.1002/ijc.33264 32818304

[10] Abdelhafeez A Ortega-Laureano L Murphy AJ Davidoff AM Fernandez-Pineda I Sandoval JA. Minimally invasive surgery in pediatric surgical oncology: practice evolution at a contemporary single-center institution and a guideline proposal for a randomized controlled study. J Laparoendosc Adv Surg Tech A. (2019) 29:1046–51. 10.1089/lap.2018.0467 31241404

[11] Sato T Kazama T Fukuzawa T Wada M Sasaki H Kudo H. Mediastinal tumor resection via open or video-assisted surgery in 31 pediatric cases: experiences at a single institution. J Pediatr Surg. (2016) 51:530–3. 10.1016/j.jpedsurg.2015.09.021 26520698

[12] Da M Peng W Mo X Fan M Wu K Sun J. Comparison of efficacy between video-assisted thoracoscopic surgery and thoracotomy in children with mediastinal tumors: 6-year experience. Ann Transl Med. (2019) 7:653. 10.21037/atm.2019.10.81 31930054 PMC6944633

[13] Acker SN Bruny JL Garrington TP Partrick DA. Minimally invasive surgical techniques are safe in the diagnosis and treatment of pediatric malignancies. Surg Endosc. (2015) 29:1203–8. 10.1007/s00464-014-3795-0 25159642

[14] Irtan S Brisse HJ Minard-Colin V Schleiermacher G Canale S Sarnacki S. Minimally invasive surgery of neuroblastic tumors in children: indications depend on anatomical location and image-defined risk factors. Pediatr Blood Cancer. (2015) 62:257–61. 10.1002/pbc.25248 25284263

[15] McDaniel JD Racadio JM Patel MN Johnson ND Kukreja K. CT-guided localization of pulmonary nodules in children prior to video-assisted thoracoscopic surgical resection utilizing a combination of two previously described techniques. Pediatr Radiol. (2018) 48:626–31. 10.1007/s00247-018-4069-0 29362842

[16] Smith TJ Rothenberg SS Brooks M Bealer J Chang J Cook BA. Thoracoscopic surgery in childhood cancer. J Pediatr Hematol Oncol. (2002) 24:429–35. 10.1097/00043426-200208000-00004 12218588

[17] Holcomb GW Tomita SS Haase GM Dillon PW Newman KD Applebaum H. Minimally invasive surgery in children with cancer. Cancer. (1995) 76:121–8. 8630863 10.1002/1097-0142(19950701)76:1<121::aid-cncr2820760119>3.0.co;2-#

[18] Bassanezi BSB Oliveira-Filho AG Miranda ML Soares L Aguiar SS. Use of BiPAP for safe anesthesia in a child with a large anterior mediastinal mass. Pediatr Anesth. (2011) 21:985–7. 10.1111/j.1460-9592.2011.03607.x 21793982

[19] Rothenberg SS Wagner JS Chang JHT Fan LL. The safety and efficacy of thoracoscopic lung biopsy for diagnosis and treatment in infants and children. J Pediatr Surg. (1996) 31:100–4. 10.1016/S0022-3468(96)90328-0 8632258

[20] Esposito C Lima M Mattioli G Mastroianni L Riccipetitoni G Monguzzi G. Thoracoscopic surgery in the management of pediatric malignancies: a multicentric survey of the Italian society of videosurgery in infancy. Surg Endosc. (2007) 21:1772–5. 10.1007/s00464-007-9246-4 17356939

[21] Sandoval C Stringel G. Video-assisted thoracoscopy for the diagnosis of mediastinal masses in children. JSLS. (1997) 1:131–3. 9876660 PMC3021275

[22] Metzelder ML Kuebler JF Shimotakahara A Glueer S Grigull L Ure BM. Role of diagnostic and ablative minimally invasive surgery for pediatric malignancies. Cancer. (2007) 109:2343–8. 10.1002/cncr.22696 17450589

[23] Spurbeck WW Davidoff AM Lobe TE Rao BN Schropp KP Shochat SJ. Minimally invasive surgery in pediatric cancer patients. Ann Surg Oncol. (2004) 11:340–3. 10.1245/ASO.2004.04.021 14993031

[24] Iwanaka T Arai M Kawashima H Kudou S Fujishiro J Imaizumi S. Endosurgical procedures for pediatric solid tumors. Pediatr Surg Int. (2004) 20:39–42. 10.1007/s00383-003-1078-2 14691638

[25] Sailhamer E Jackson CC Vogel AM Kang S Wu Y Chwals WJ. Minimally invasive surgery for pediatric solid neoplasms. Am Surg. (2003) 69:566–8. 12889617

[26] Holcomb GW. Minimally invasive surgery for solid tumors. Semin Surg Oncol. (1999) 16:184–92. 10.3390/children5120158 9988872

[27] Saenz NC Conlon KC Aronson DC LaQuaglia MP. The application of minimal access procedures in infants, children, and young adults with pediatric malignancies. J Laparoendosc Adv Surg Tech A. (1997) 7:289–94. 10.1089/lap.1997.7.289 9453873

[28] Rodgers BM Moazam F Talbert JL. Thoracoscopy in children. Ann Surg. (1979) 189:176–80. 10.1097/00000658-197902000-00008 311621 PMC1397035

[29] Fraga JC Aydogdu B Aufieri R Silva GVM Schopf L Takamatu E. Surgical treatment for pediatric mediastinal neurogenic tumors. Ann Thorac Surg. (2010) 90:413–8. 10.1016/j.athoracsur.2010.04.086 20667322

[30] Fraga JC Rothenberg S Kiely E Pierro A. Video-assisted thoracic surgery resection for pediatric mediastinal neurogenic tumors. J Pediatr Surg. (2012) 47:1349–53. 10.1016/j.jpedsurg.2012.01.067 22813795

[31] Malek MM Mollen KP Kane TD Shah SR Irwin C. Thoracic neuroblastoma: a retrospective review of our institutional experience with comparison of the thoracoscopic and open approaches to resection. J Pediatr Surg. (2010) 45:1622–6. 10.1016/j.jpedsurg.2010.03.018 20713210

[32] Liu YW Chen HW Lee JY Chiang HH Li HP Chang PC. Is a chest tube necessary after video-assisted thoracoscopic mediastinal tumor resection? Thorac Cardiovasc Surg. (2019) 69:181–8. 10.1055/s-0039-1683879 30934095

[33] Malkan AD Loh AHP Fernandez-Pineda I Sandoval JA. The role of thoracoscopic surgery in pediatric oncology. J Laparoendosc Adv Surg Tech A. (2014) 24:819–26. 10.1089/lap.2014.0252 25290585

[34] Pentek F Schulte JH Schweiger B Metzelder M Schündeln MM. Development of port-site metastases following thoracoscopic resection of a neuroblastoma. Pediatr Blood Cancer. (2015) 63:149–51. 10.1002/pbc.25677 26206749

[35] Christison-Lagay ER Thomas D. Minimally invasive approaches to pediatric solid tumors. Surg Oncol Clin N Am. (2019) 28:129–46. 10.1016/j.soc.2018.07.005 30414678

[36] Gurria JP Malek MM Heaton TE Gehred A Lautz TB Rhee DS. Minimally invasive surgery for abdominal and thoracic neuroblastic tumors: a systematic review by the APSA cancer committee. J Pediatr Surg. (2020) 55:2260–72. 10.1016/j.jpedsurg.2020.02.019 32151400

[37] Sánchez-Sánchez Á Girón-Vallejo Ó Ruiz-Pruneda R Fernandez-Ibieta M García-Calderon D Villamil V. Three-Dimensional printed model and virtual reconstruction: an extra tool for pediatric solid tumors surgery. European J Pediatr Surg Rep. (2018) 06:e70–6. 10.1055/s-0038-1672165 30370204 PMC6202581

[38] Smelt JLC Suri T Valencia O Jahangiri M Rhode K Nair A. Operative planning in thoracic surgery: a pilot study comparing imaging techniques and three-dimensional printing. Ann Thorac Surg. (2019) 107:401–6. 10.1016/j.athoracsur.2018.08.052 30316856

[39] Kiraly L Tofeig M Jha NK Talo H. Three-dimensional printed prototypes refine the anatomy of post-modified Norwood-1 complex aortic arch obstruction and allow presurgical simulation of the repair. Interact CardioVasc Thorac Surg. (2015) 22:238–40. 10.1093/icvts/ivv320 26590304

[40] Barsness KA Rooney DM Davis LM. Collaboration in simulation: the development and initial validation of a novel thoracoscopic neonatal simulator. J Pediatr Surg. (2013) 48:1232–8. 10.1016/j.jpedsurg.2013.03.015 23845612

[41] Shiraishi I Yamagishi M Hamaoka K Fukuzawa M Yagihara T. Simulative operation on congenital heart disease using rubber-like urethane stereolithographic biomodels based on 3D datasets of multislice computed tomography. Eur J Cardiothorac Surg. (2010) 37:302–6. 10.1016/j.ejcts.2009.07.046 19758813

[42] Aiolfi A Nosotti M Micheletto G Khor D Bonitta G Perali C. Pulmonary lobectomy for cancer: systematic review and network meta-analysis comparing open, video-assisted thoracic surgery, and robotic approach. Surgery. (2021) 169:436–46. 10.1016/j.surg.2020.09.010 33097244

[43] Tabone MD Kalifa C Rodary C Raquin M Valteau-Couanet D Lemerle J. Osteosarcoma recurrences in pediatric patients previously treated with intensive chemotherapy. J Clin Oncol. (1994) 12:2614–20. 10.1200/JCO.1994.12.12.2614 7989936

[44] Lautz TB Farooqui Z Jenkins T Heaton TE Doski JJ Cooke-Barber J. Thoracoscopy vs thoracotomy for the management of metastatic osteosarcoma: a pediatric surgical oncology research collaborative study. Int J Cancer. (2020) 148:1164–71. 32818304 10.1002/ijc.33264

[45] Sartorelli KH Partrick D Meagher DP Jr. Port-site recurrence after thoracoscopic resection of pulmonary metastasis owing to osteogenic sarcoma. J Pediatr Surg. (1996) 31:1443–4. 10.1016/S0022-3468(96)90852-0 8906685

[46] Meehan JJ. Robotic surgery for pediatric tumors. Cancer. (2013) 19:183–8. 10.1097/PPO.0b013e318289486c 23528728

[47] van Dalen EC de Lijster MS Leijssen LG Michiels EM Kremer LC Caron HN. Minimally invasive surgery versus open surgery for the treatment of solid abdominal and thoracic neoplasms in children. Cochrane Database Syst Rev. (2015) 1:CD008403. 10.1002/14651858.CD008403.pub3 25560834 PMC7180085

[48] Blanc T Meignan P Vinit N Ballouhey Q Pio L Capito C. Robotic surgery in pediatric oncology: lessons learned from the first 100 tumours-A nationwide experience. SSRN J [Preprint]. (2020). Available at: https://papers.ssrn.com/sol3/papers.cfm?abstract_id=3757404 (accessed September 5, 2021).

[49] Blanc T Pio L Clermidi P Muller C Orbach D Minard-Colin V. Robotic-assisted laparoscopic management of renal tumors in children: preliminary results. Pediatr Blood Cancer. (2019) 66 (Suppl. 3):e28431. 10.1002/pbc.27867 31136081

[50] Mattioli G Pini Prato A Razore B Leonelli L Pio L Avanzini S. Da Vinci robotic surgery in a pediatric hospital. J Laparoendosc Adv Surg Tech. (2017) 27:539–45. 10.1089/lap.2016.0390 28278402

[51] Giordano SH Kuo YF Duan Z Hortobagyi GN Freeman J Goodwin JS. Limits of observational data in determining outcomes from cancer therapy. Cancer. (2008) 112:2456–66. 10.1002/cncr.23452 18428196 PMC3856661

